# Small Bowel Volvulus Secondary to a Mesenteric Cystic Lymphangioma: A Case Report and Literature Review

**DOI:** 10.7759/cureus.100270

**Published:** 2025-12-28

**Authors:** Dimitrios K Vlachos, Panagiotis Dorovinis, Stylianos Kykalos, Nicolaos Machairas

**Affiliations:** 1 2nd Department of Propaedeutic Surgery, Laiko General Hospital of Athens, Athens, GRC

**Keywords:** abdominal lymphangioma, mesenteric cyst, mesenteric cystic lymphangioma, mesenteric volvulus, small bowel ischemia, small bowel obstruction, small bowel volvulus

## Abstract

Mesenteric cystic lymphangioma (MCL) is a rare, benign lymphatic malformation that typically occurs in children and is infrequently encountered in adults. Although most cases are asymptomatic, complications such as volvulus, intracystic hemorrhage, or compression of adjacent structures may lead to acute abdominal presentations. We report the case of a 39-year-old male who presented with vomiting, abdominal pain, and absence of bowel movement for 48 hours. Imaging revealed a large multiloculated mesenteric cystic lesion associated with small bowel volvulus and signs of ischemia. Emergency laparotomy demonstrated a multicystic mass arising from the ileal mesentery, causing mesenteric rotation and obstruction. En bloc resection of the affected bowel segment and the mass was performed, followed by primary anastomosis. The postoperative course was uneventful. Histopathology confirmed a cystic lymphangioma without evidence of malignancy. MCL in adults is uncommon, and presentation with small bowel volvulus is exceptionally rare. Diagnosis is based primarily on imaging, with a CT scan being the modality of choice. Complete surgical resection remains the gold standard treatment, as incomplete excision is associated with high recurrence rates. Although rare, MCL should be included in the differential diagnosis of newly detected mesenteric cystic masses in adults. Early recognition and complete surgical excision are essential to prevent complications such as volvulus and to minimize recurrence.

## Introduction

Cystic lymphangiomas (CL) are rare, benign malformations of the lymphatic system found mainly in children [1]. Although the etiology remains unclear, it is thought that there is a congenital background, mainly supported by the correlation of the disease with its early onset [1]. CL are most commonly found in the head, neck, and axillary region in both children and adults [2]. Abdominal CLs are quite rare and represent less than 5% of all CL cases, with mesentery being the most frequent abdominal location with an estimated incidence of 1/100000 in adults and 1/20000 in children [3]. Mesenteric CLs (MCLs) are usually asymptomatic and can rarely present with diverse symptoms, most commonly with vague abdominal pain [4]. It is important not to confuse cystic lymphangiomas with lymphangiomatosis, which presents more frequently in adolescence or early adulthood and is characterized by the development of multifocal or multisystem lymphangiomas. 

Although most mesenteric cystic lymphangiomas remain asymptomatic and are often discovered incidentally, acute presentations due to complications have been reported. These include infection, intracystic hemorrhage, and, rarely, intestinal obstruction [4]. Volvulus represents one of the most severe complications, as it may lead to bowel ischemia and necessitate emergency surgical intervention. Due to the rarity of this entity, data regarding the exact incidence of volvulus and associated mortality are limited. 

In this report, we present the case of a 39-year-old male admitted to our department due to small bowel volvulus secondary to an MCL of the terminal ileum.

## Case presentation

This case report was prepared in accordance with the CARE (Consolidated Reporting Standards for Case Reports) guidelines.

**Figure 1:**
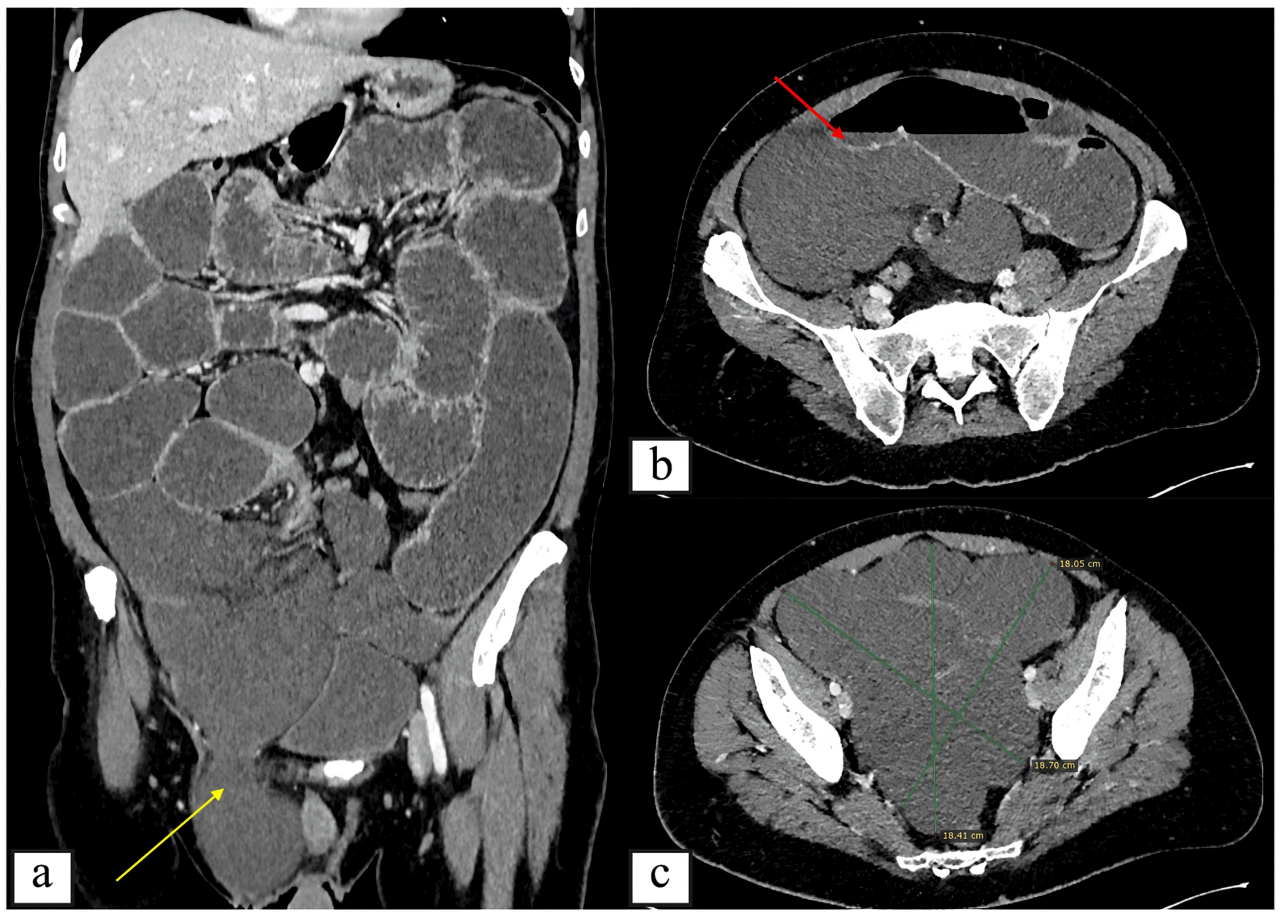
Contrast-enhanced CT images of the abdomen (a) Coronal CT image demonstrating a large multicystic lesion in the lower right abdomen protruding into a right inguinal hernia (yellow arrow). (b) Axial (transverse) CT images demonstrating differences in enhancement between the small bowel wall (red arrow) and the cystic lesion, as well as the overall size of the multiloculated mass measuring approximately 18.3 × 18.7 × 18.4 cm. (c) Axial contrast-enhanced CT image demonstrating the overall size and extent of the large multiloculated cystic mass, measuring approximately 18.3 × 18.7 × 18.4 cm.

A 39-year-old Caucasian male patient, with a history of schizophrenia well-controlled under treatment, presented to the Emergency Department of our hospital due to multiple episodes of vomiting, abdominal pain, and absence of bowel movement for the past 48 hours. On physical examination, he demonstrated abdominal distention and tenderness, particularly in the lower abdomen. Bowel sounds were hypoactive. A soft, non-tender mass was palpated in the right inguinal area, most probably representing a right inguinal hernia, without signs of inflammation or strangulation. Abdominal X-ray revealed multiple air-fluid levels and dilated loops of small intestine, consistent with small bowel obstruction. The patient was hypotensive and tachycardic (110 bpm) and was resuscitated with IV fluids and subsequently underwent an abdominal computer tomography (CT) scan with IV contrast (Figure 1). The study revealed an intraperitoneal multilobulated cystic lesion without enhancement of the cystic wall or the septae arising from the ileal mesentery, rotation of the small bowel mesentery, as well as thickened small bowel walls suggestive of ischemia.

**Figure 2:**
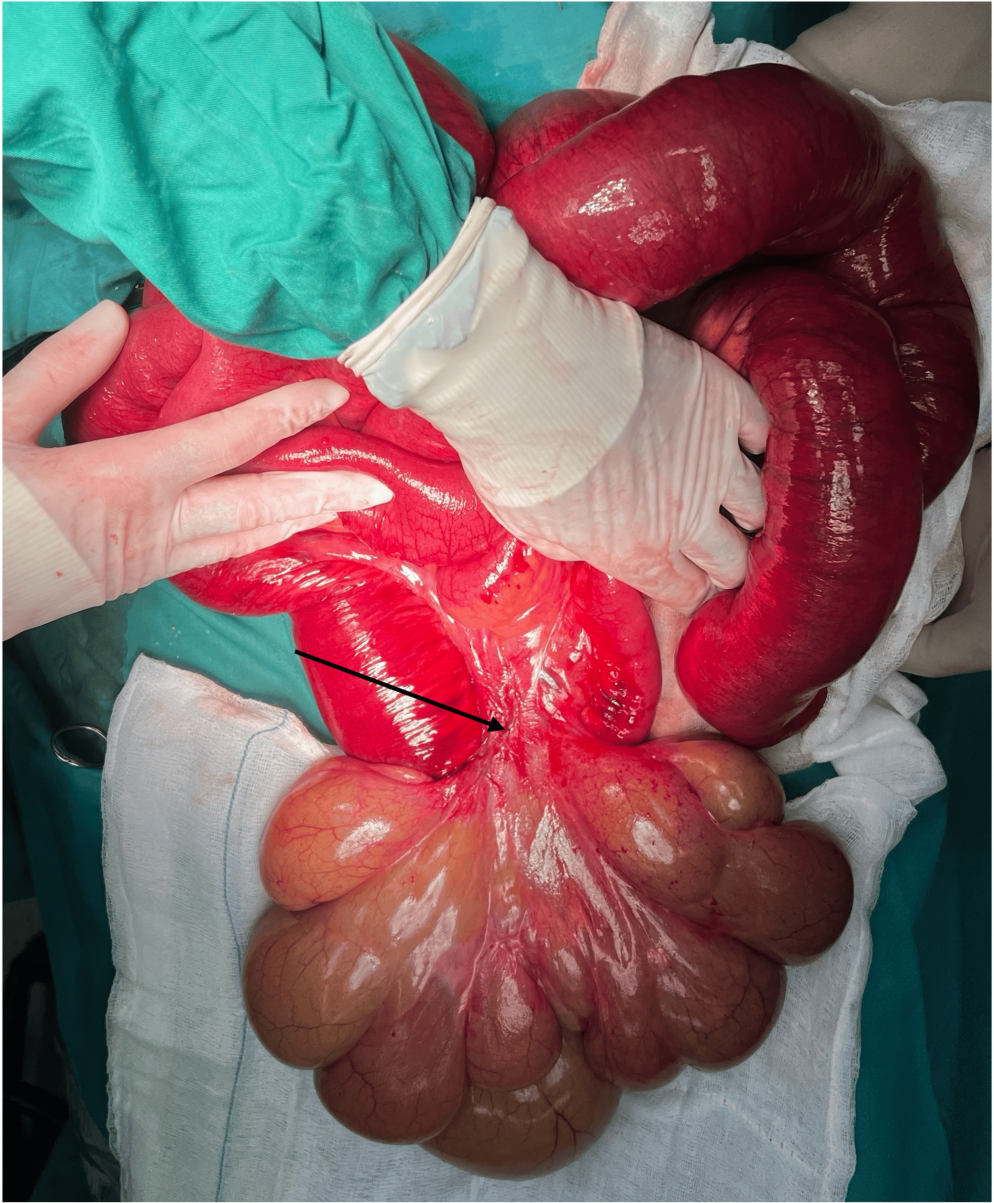
The large multicystic lesion arising from the mesentery of the ileum (black arrow).

Consequently, the patient was transferred to the operating room for an emergency exploratory laparotomy. A large multicystic mass originating from the ileal mesentery was demonstrated as the causative factor for ileal volvulus (Figure 2).

The mass had seemingly caused a rotation of the mesentery for more than 180° and had caused obstruction and ischemia of the adjacent ileal loop. The affected small bowel was subsequently resected along with the voluminous multicystic mesenteric mass, and a hand-sewn side-to-side anastomosis was performed. No repair of the right inguinal hernia defect was performed during the emergency procedure due to the contaminated surgical field and the priority given to bowel resection and restoration of intestinal continuity. The patient recovered uneventfully, with resolution of symptoms and normalization of bowel function within 24 hours. He was discharged on the fifth postoperative day. Six months postoperatively, he remains in excellent clinical condition, with no signs of recurrence based on physical examination and radiologic work-up.

Histopathologic analysis of the resected specimen revealed a large (18.3×16×17.2 cm) MCL composed of many dilated and cystic lymphatic vessels lined with flattened endothelial cells, thus confirming the diagnosis of mesenteric cystic lymphangioma. No signs of atypia or malignancy were noted. Immunochemistry was positive for CD31, CD34, and D2-40.

## Discussion

Cystic lymphangioma (CL) is a rare, benign malformation of the lymphatic vessels, and malignant transformation has been reported only exceptionally [1]. Regarding its etiology, the most widely accepted hypothesis is a congenital origin, specifically abnormal embryonic development of the lymphatic system leading to sequestration of lymphatic vessels [5]. Other proposed contributing factors include inflammation, abdominal trauma, prior abdominal surgery, radiation exposure, or lymphatic obstruction [5]. As a congenital malformation, it is most commonly diagnosed in children, with more than 80% of cases identified during the first year of life [6]. CL can potentially affect any organ except the central nervous system, which lacks lymphatic vessels [1]. They most frequently occur in the head, neck, or axilla, whereas the abdomen is an uncommon primary location [2]. Abdominal cystic lymphangiomas arise mainly from the mesentery, followed by the greater omentum, mesocolon, and retroperitoneum [4].

**Figure 3:**
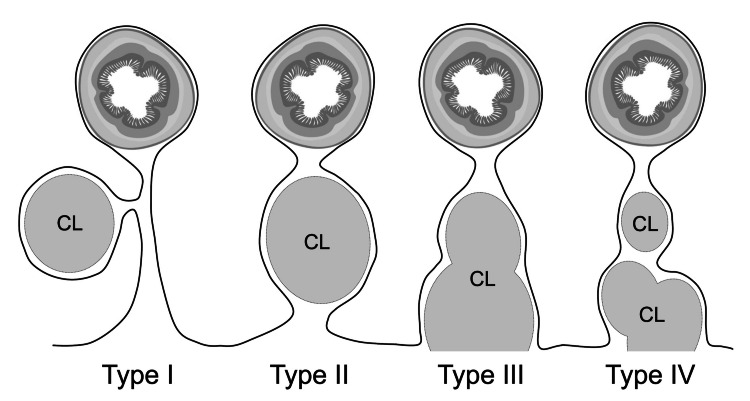
Classification of mesenteric cystic lymphangiomas into four types Diagram showing the four types of mesenteric cystic lymphangiomas: Type I (pedicled MCL prone to intestinal volvulus), Type II (sessile MCL within mesenteric boundaries), Type III (MCL with retroperitoneal extension), and Type IV (multicentric MCLs). CL: cystic lymphangiomas; MCL: mesenteric cystic lymphangiomas Source: Schematic illustration created by the authors based on the original description by Losanoff et al. [11].

Mesenteric cystic lymphangiomas (MCLs) are usually asymptomatic and discovered incidentally. However, they may be clinically challenging because, when symptomatic, their presentation is generally nonspecific. Abdominal pain and a palpable mass are the most common findings, whereas complications such as rupture, infection, intracystic hemorrhage, volvulus, or compression of adjacent structures are less common but may present as an acute abdomen [1]. Losanoff et al. proposed a classification system for MCLs, categorizing them into four types, as shown in Figure 3 [11].

Our patient presented with a Type I MCL, which is more prone to causing mesenteric malrotation and subsequent small bowel obstruction and ischemia. To the best of our knowledge, MCL-induced volvulus is a very rare presentation, with only a limited number of cases reported in the literature [7-11].

Because their symptomatology is highly variable, the diagnosis of MCL relies primarily on imaging. Abdominal ultrasound is an excellent initial modality, typically revealing a thin-walled, multiloculated cystic lesion with anechoic content and no calcifications [12]. In cases of intracystic hemorrhage, echogenic fluid and Doppler flow signals may be observed. Contrast-enhanced CT remains the gold standard for diagnosis, as it provides detailed information regarding the location, morphology, size, and relationship of the lesion to adjacent structures [12]. MCL typically appears as a uni- or multiloculated cystic mass without significant enhancement of the walls or septae [8]. MRI is particularly useful for characterizing the cystic content [12]. Differential diagnoses include other intra-abdominal cystic lesions such as mesenteric cysts, abdominal lymphomas, metastatic disease, tuberculosis, hydatid cysts, small bowel adenocarcinoma, and rare mesenteric tumors such as desmoid tumors, schwannomas, smooth muscle tumors, sarcomas, cystic mesotheliomas, lymphangiosarcomas, and lymphangiomas with myxoid degeneration [13].

Surgical resection remains the gold standard for the treatment of MCL and provides a definitive diagnosis through histopathological evaluation. Radical excision, while preserving adjacent organs and structures whenever possible, is the optimal approach. Recurrence rates may reach 40% when complete resection is not achieved, whereas en bloc resection with clear margins reduces recurrence to approximately 17% [13]. A spontaneous regression rate of 1.6-16% has been described [14]. Both laparoscopic and open techniques can be used safely, although in cases of large lesions, aspiration of cystic content during laparoscopy may facilitate removal; however, caution is required to avoid spillage, especially in lesions that may mimic CL [4]. In our case, the markedly distended abdomen secondary to volvulus and the large size of the lesion necessitated an open approach. Aspiration with or without a sclerosing agent may be considered for complicated or unresectable lesions, but recurrence rates approach 100% [2]. More recent studies have proposed targeted therapies using lymphatic markers such as D2-40, Prox-1, VEGFR-3, PDGFs, and Ki-67, which may be promising for unresectable or recurrent cases [15].

Definitive diagnosis of MCL is established through histopathology, which characteristically shows dilated lymphatic vessels lined by flattened endothelial cells without atypia and surrounded by abundant lymphoid tissue [16]. Immunohistochemistry is typically positive for CD31, CD34, CD45, factor VIII-related antigen, HMB-45, D2-40, and calretinin [8].

In our case, part of the mesenteric cystic lymphangioma was noted to protrude into a right inguinal hernia. Although the primary mechanism of obstruction and volvulus was mesenteric rotation caused by the mass, incarceration of part of the lesion within the inguinal canal may have contributed to the development of volvulus. This represents an unusual presentation of an inguinal hernia containing a mesenteric cystic lesion.

## Conclusions

Mesenteric cystic lymphangioma is a rare pathological entity. Its clinical and radiological presentation is diverse and nonspecific, making preoperative diagnosis challenging. Even in adults, mesenteric cystic lymphangioma should be included in the differential diagnosis of newly identified mesenteric cystic lesions. Total surgical excision remains the optimal treatment, with low recurrence rates, while also addressing rare but potentially serious complications such as small bowel volvulus. Definitive diagnosis relies on histopathological examination. Given the rarity of MCLs, further research is needed to explore the potential of targeted therapies, particularly in cases that demonstrate recurrent or aggressive behavior.
